# Influence of Sintering Parameters on the Mechanical Behaviour of Lithium Disilicate Glass Ceramics: An In-Vitro Study

**DOI:** 10.3390/jfb16110408

**Published:** 2025-11-02

**Authors:** Mai Soliman, Raghad Alotaibi, Abrar Almutairi, Asma Alzahrani, Reem Abunyan, Aseel Rozi, Dalia Alamri, Shahad Almakenzi, Elzahraa Eldwakhly, Alhanoof Aldegheishem

**Affiliations:** 1 Department of Clinical Dental Sciences, College of Dentistry, Princess Nourah bint Abdulrahman University, P.O. Box 84428, Riyadh 11671, Saudi Arabia; eaeldwakhly@pnu.edu.sa (E.E.); asaldegheishem@pnu.edu.sa (A.A.); 2 College of Dentistry, Princess Nourah bint Abdulrahman University, P.O. Box 84428, Riyadh 11671, Saudi Arabia; 439003069@pnu.edu.sa (R.A.); 438001338@pnu.edu.sa (A.A.); 439000640@pnu.edu.sa (A.A.); 439001917@pnu.edu.sa (R.A.); 439005535@pnu.edu.sa (A.R.); 439003535@pnu.edu.sa (D.A.); 439002502@pnu.edu.sa (S.A.)

**Keywords:** lithium disilicate, translucency, sintering, surface roughness, fracture load

## Abstract

This study investigates the effect of different sintering parameters on the surface roughness and fracture resistance of different CAD/CAM lithium disilicate ceramics before and after thermocycling and simulated toothbrushing. Sixty lithium disilicate ceramic samples were categorized according to ceramic type (AMB: Amber Mill, ECAD: IPS e.max CAD) and level of translucency (LT: low and HT: high) into four groups: AMB_LT, AMB_HT, ECAD_LT and ECAD_HT. Specimens were prepared to 2 mm thickness, crystallized, polished, and subjected to thermocycling to simulate five years of clinical aging. Simulated toothbrushing was performed using a soft-bristled mechanical brushing system under controlled force and strokes. Surface roughness was assessed using a profilometer before and after brushing, and fracture resistance was measured using a universal testing machine. Data were statistically evaluated using paired *t*-tests, one-way ANOVA with Bonferroni post hoc correction (*p* ≤ 0.05). LT lithium disilicate specimens exhibited significantly smoother surfaces than high-translucency counterparts. After thermocycling and brushing simulation, all groups showed a statistically significant increase in surface roughness, ranging between 0.239 ± 0.012 μm (AMB_LT) and 0.486 ± 0.014 μm (ECAD_HT). In terms of fracture resistance, the highest values were recorded for ECAD_HT (636 ± 8.29 N), and the lowest in the AMB_HT group (546.3 ± 21.9 N) with significant differences observed between materials and translucency levels. Sintering parameters and artificial aging significantly influenced the surface roughness and fracture resistance of lithium disilicate ceramics. Low-translucency variants demonstrated smoother surfaces and higher strength than high-translucency groups, while all materials exhibited increased roughness after aging. These findings provide guidance for the selection of lithium disilicate ceramics, balancing esthetic and mechanical requirements in anterior and posterior restorations.

## 1. Introduction

Ceramics have become the restorative material of choice in dentistry due to their excellent esthetic properties, chemical inertness, and biocompatibility [1]. Glass ceramics, in particular, are widely used because of their favorable physical and chemical characteristics, such as translucency, low thermal conductivity, biocompatibility, adequate strength, wear resistance, and chemical durability [1,2]. Over recent decades, significant research and development have been directed toward improving the mechanical performance and processing technologies of dental ceramics, leading to their widespread clinical use [3,4].

Lithium disilicate (LD) was first introduced as a core material in the late 1990s (IPS Empress II, Ivoclar Vivadent) and has since evolved into one of the most popular glass-ceramics in restorative dentistry [5,6]. Owing to its excellent strength, machinability, and superior translucency, LD is extensively used in veneers, inlays, onlays, anterior crowns, as well as posterior crowns and short-span fixed dental prostheses [7,8,9,10]. Its hardness, while higher than enamel, is relatively close to natural tooth structure compared to other ceramics, making it suitable for long-term clinical applications [2,9,11]. In general, glass ceramics such as lithium disilicate exhibit a biphasic microstructure composed of a residual glassy matrix and a dispersed crystalline phase. The glassy phase governs translucency and esthetic appearance by transmitting and scattering light, whereas the crystalline phase enhances mechanical performance through crack deflection and toughening mechanisms. The size, orientation, and volume fraction of lithium disilicate crystals therefore determine the balance between optical and mechanical properties, explaining the typical trade-off between higher translucency and lower strength in these materials [12].

Lithium disilicate can be fabricated either by heat-pressing or CAD/CAM milling techniques. In the conventional press technique, lithium disilicate is supplied in ingot form and processed using the lost-wax method, whereas CAD/CAM blocks, such as IPS e.max CAD, are provided in a partially crystallized state (the so-called *blue stage*). This allows for efficient and less tool-intensive milling, followed by a crystallization firing to achieve the final lithium disilicate structure with full strength and optical properties [10,13,14,15]. The increasing integration of CAD/CAM systems into daily practice—facilitated by advances in intraoral scanners, design software, and milling machines—has enabled dentists to fabricate highly precise and predictable restorations with greater efficiency [16,17].

New lithium disilicate materials, including Amber Mill and Amber Press, have been introduced with high esthetic potential, machinability, and edge stability [18,19]. Processing parameters, particularly sintering temperature and holding time, play a crucial role in determining the microstructure, surface quality and mechanical performance of these ceramics [20,21,22,23]. Optimized sintering can enhance crystallization, fracture resistance, and flexural strength, whereas inappropriate parameters may lead to surface roughness, coarsened microstructure, and compromised esthetics. In addition, intraoral aging factors such as thermal fluctuations and repeated mechanical wear, including toothbrushing, can further modify surface roughness and mechanical reliability [24,25,26,27]. These considerations underscore the clinical importance of evaluating both material composition and processing conditions.

Therefore, the aim of this in vitro study was to investigate the influence of different sintering parameters on the fracture resistance and surface roughness of two different lithium disilicate ceramic materials. The null hypotheses were that (1) sintering parameters do not influence the surface roughness and fracture resistance of lithium disilicate; and (2) thermocycling and simulated toothbrushing do not affect surface roughness and fracture resistance of these materials.

## 2. Materials and Methods

For this comparative in vitro study, a total of 60 lithium disilicate ceramic disks (10 mm diameter × 2 mm thickness) were fabricated according to ISO 6872:2015 [28] from different CAD/CAM lithium disilicate brands and divided according to ceramic type and level of translucency into four groups (n = 15) (Table 1).

The sintering and crystallization processes of all groups were carried out according to manufacturer’s recommendations. Samples in groups AMB_LT and AMB_HT were predried at 400 °C prior to crystallization. Heat treatment was performed using translucency-specific protocols recommended by the manufacturer, where AMB_HT was heated at 815 °C for 15 min plus a 21.5 min vacuum hold, and AMB_LT at 840 °C for 22.2 min. ECAD_LT and ECAD_HT were crystallized following the manufacturer’s standard Programat furnace cycle (P300, P500, P700). The firing program consisted of initial predrying at 403 °C for 6 min, followed by heating at a rate of 60 °C/min up to 770 °C, held for 10 min; subsequent heating at 30 °C/min to a final crystallization temperature of 850 °C, with a holding time of 10 min. Controlled cooling was performed to 700 °C before returning to room temperature. This cycle applies to both ECAD_LT and ECAD_HT ensuring complete crystallization of lithium disilicate (Table 2).

Following crystallization, all disks were polished with a fully automated polishing unit (Automata, Ried, Germany) using standardized sequential abrasive protocols. Subsequently, they were ultrasonically cleaned in deionized water to remove residual debris and surface contaminants prior to testing.

Thereafter, initial surface roughness (Ra) was determined using a non-contact profilometer (Bruker, Bremen, Germany). For each specimen, measurements were taken at multiple predefined locations, and the mean Ra value was calculated in micrometers (µm). These measurements served to establish baseline surface roughness prior to aging simulations.

**Figure 1:**
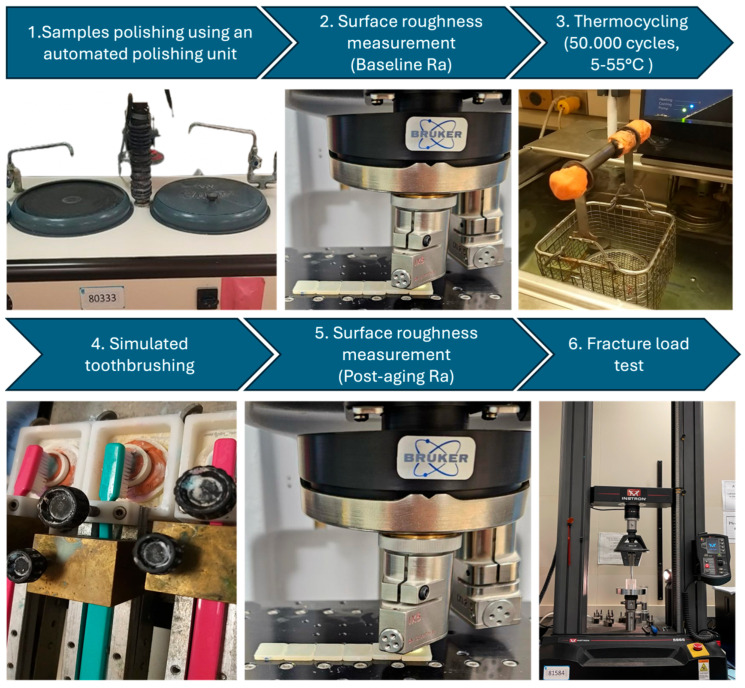
An image showing the different steps of the in vitro investigation, starting with polishing of the ceramic samples, baseline surface roughness measurements followed by thermocycling and simulated toothbrushing, then post-aging surface roughness measurements and fracture resistance.

Specimens were subjected to 50,000 thermocycles to simulate approximately five years of clinical aging (THE1400 thermocycler, SD Mechatronics, Feldkirchen-Westerham, Germany). Cycling was performed between cold (5 °C) and hot (55 °C) water baths in a standard thermocycling chamber, with a dwell time of 30 s in each bath and a transfer time of 5 s. After thermocycling, specimens underwent simulated toothbrushing using a soft-bristled mechanical brushing machine (ZM3 toothbrush simulator, SD Mechatronics, Germany), calibrated to represent approximately five years of clinical use. The brushing simulation was performed under a constant vertical load of 200 g with a stroke frequency of 120 strokes/min, totaling 75,000 double strokes (150,000 cycles) per specimen. Brushing was conducted in a slurry of toothpaste and deionized water (1:2 *w*/*v*) to mimic intraoral conditions. All samples were brushed under identical conditions to ensure consistency (Figure 1).

Following aging, surface roughness was assessed using a non-contact optical profilometer (Bruker, Germany). Each specimen was stabilized to prevent movement, and three predefined, centrally distributed points were scanned with a laser-based sensor, avoiding edges. The profilometer recorded average surface roughness (Ra) values in micrometers (µm), representing vertical deviations from an ideal smooth reference plane. Measurements were conducted under standardized ambient conditions and identical calibration settings as in the baseline evaluation. For each specimen, the mean Ra value was calculated from the three scanned regions and used for statistical analysis to evaluate the effects of the aging procedures.

**Figure 2:**
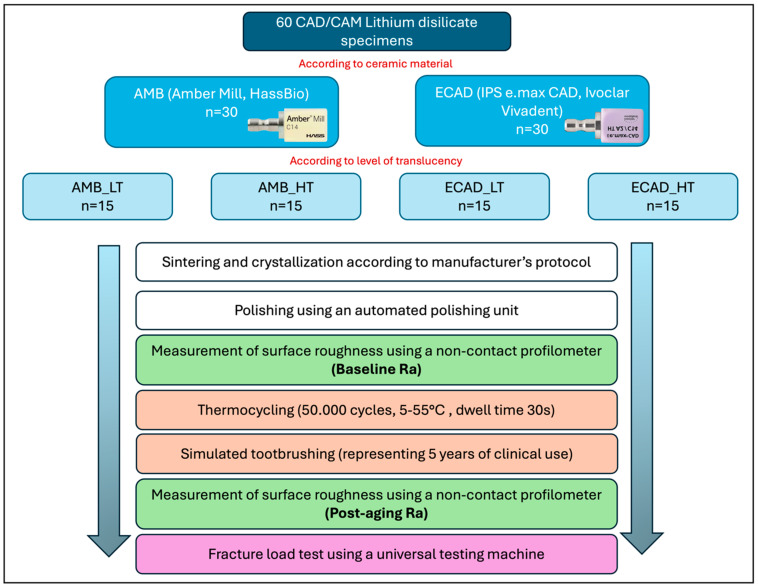
A detailed workflow of the in vitro investigation.

After thermocycling and simulated toothbrushing, fracture resistance testing was performed to evaluate the specimens’ ability to withstand functional occlusal forces. Tests were conducted using a universal testing machine (Instron, Norwood, MA, USA), calibrated for precise load application and detection. Each specimen was positioned on a flat, custom-made metal support base to ensure stability during testing. A 4 mm spherical stainless-steel indenter was aligned centrally with the specimen’s long axis to promote uniform stress distribution, simulating functional intraoral loading conditions. A monotonic compressive load was applied vertically at a crosshead speed of 1 mm/min until catastrophic failure, defined as visible fracture or audible cracking. The machine automatically recorded the maximum load (N) sustained at fracture. All specimens were tested under identical environmental conditions using the same alignment jig to ensure consistency across groups. Fracture resistance values were documented for each specimen and used for statistical comparisons between material types and translucency groups, providing a direct measure of mechanical resilience under high-stress conditions. Figure 2 represents the different test groups and the workflow of the study.

All collected data were analyzed using IBM SPSS Statistics version 20.0. The Shapiro–Wilk test was applied to assess the normality of the data distribution. Surface roughness and fracture resistance values were expressed as mean ± standard deviation (SD) for each group. Paired *t*-tests were performed to compare pre- and post-aging surface roughness values within groups, while one-way ANOVA with Bonferroni post hoc correction was used for intergroup comparisons. To evaluate the effect of material type while accounting for baseline differences, Analysis of Covariance (ANCOVA) was applied. A significance level of *p* ≤ 0.05 was considered for all statistical tests.

## 3. Results

The effect of sintering parameters on surface roughness showed that AMB_LT had significantly lower roughness (0.239 ± 0.012 μm) compared to AMB_HT (0.432 ± 0.046 μm) (*p* ≤ 0.05). When compared with other groups, AMB_LT exhibited lower roughness than ECAD_LT (0.375 ± 0.054 μm), and AMB_HT was smoother than ECAD_HT (0.486 ± 0.014 μm), indicating that AMB materials generally produced smoother surfaces than ECAD groups regardless of sintering conditions (Table 3).

**Figure 3:**
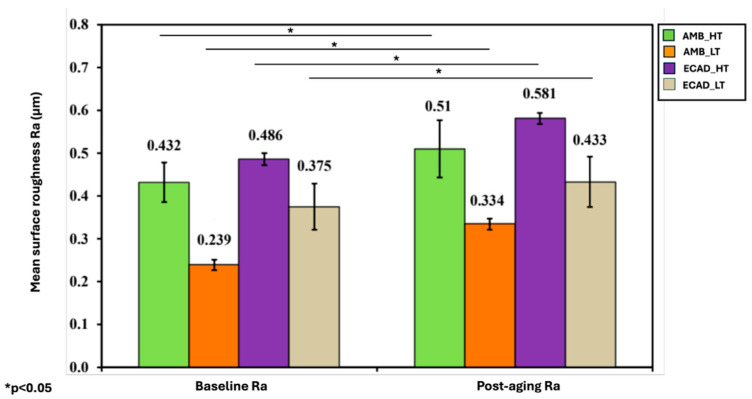
Mean surface roughness of the ceramic samples among the four investigated groups at baseline and after thermocycling and toothbrushing. “*” indicates statistically significant differences (*p* < 0.05).

Thermocycling and simulated toothbrushing resulted in a statistically significant increase in surface roughness across all groups (*p* ≤ 0.05), with all materials showing higher roughness values after thermocycling (Figure 3).

Regarding fracture resistance, AMB_LT demonstrated significantly higher values (568 ± 14.73 N) than AMB_HT (546.3 ± 21.90 N). ECAD_LT exhibited the highest fracture resistance (636 ± 8.29 N), followed by ECAD_HT (566.4 ± 10.65 N), both of which were significantly higher than their AMB counterparts. The overall trend in fracture resistance was ECAD_LT followed by ECAD_HT, AMB_LT and AMB_HT (Table 4).

**Figure 4:**
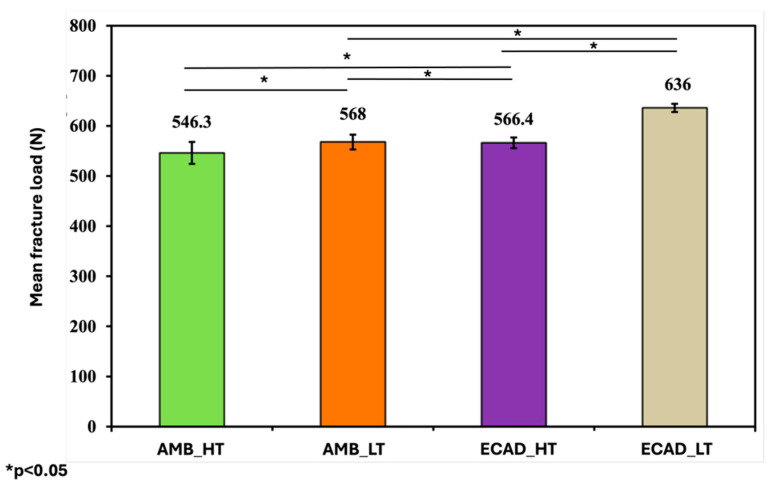
Mean fracture load values of the ceramic samples among the four investigated groups in Newton (N). “*” indicates statistically significant differences (*p* < 0.05).

Statistical analyses using one-way ANOVA with post hoc testing confirmed significant differences among the materials in both surface roughness and fracture resistance, while paired *t*-tests verified the effect of thermocycling on increasing surface roughness (Figure 4).

## 4. Discussion

This study evaluated the influence of sintering parameters, translucency levels, and artificial aging on the surface roughness and fracture resistance of lithium disilicate CAD/CAM ceramics. The null hypotheses were that (1) sintering parameters do not influence the surface roughness and fracture resistance of lithium disilicate, and (2) thermocycling and simulated toothbrushing do not affect surface roughness and fracture resistance of these materials. Based on the present findings, both hypotheses were rejected.

Two lithium disilicate systems were examined: IPS e.max CAD, supplied in a partially crystallized state, and Amber Mill, a newer lithium disilicate glass ceramic that allows translucency adjustment via firing temperature. At baseline, both materials showed distinct surface roughness profiles depending on translucency. LT ceramics exhibited smoother surfaces than their HT counterparts, with Amber Mill LT presenting the lowest Ra values. This difference is likely related to the unique firing protocol of Amber Mill, which modifies translucency by controlling crystal growth and distribution. In contrast, IPS e.max CAD relies on a standardized crystallization cycle, producing a relatively coarser microstructure at higher translucency levels. These material-specific processing approaches therefore contribute to the observed variation in surface topography and support rejection of the first hypothesis.

The differences observed between the two lithium disilicate ceramics can be attributed also to variations in their microstructural composition and crystal morphology. IPS e.max CAD is composed of approximately 70% needle-like lithium disilicate crystals embedded in a glassy matrix, while Amber Mill exhibits a finer and more homogeneous crystal distribution with a higher glass content in the pre-crystallized state. During sintering, translucency modifications—such as those used to obtain high- and low-translucency variants—are achieved by altering crystal size, crystal-to-glass ratio, and the amount of residual porosity [29,30]. Higher translucency (HT) ceramics generally contain smaller or fewer crystals and a more silica-rich glass phase, improving light transmission but reducing mechanical integrity due to diminished crack deflection, lower energy dissipation at the crystal–matrix interface, and increased susceptibility to microcrack initiation Conversely, ceramics with lower translucency (LT) maintain a denser microstructure with higher crystalline content, enhancing resistance to crack propagation and surface wear [31,32]. Therefore, the greater roughness and reduced fracture resistance observed in highly translucent groups are consistent with the expected trade-off between optical and mechanical properties governed by microstructural architecture. These results indicate that translucency modifications, while beneficial for optical performance, may compromise mechanical behavior, highlighting the importance of balancing esthetic and functional requirements in lithium disilicate restorations.

Following thermocycling and brushing simulation, all groups exhibited a statistically significant increase in surface roughness (*p* < 0.001). Amber Mill LT maintained relatively smoother surfaces compared to other groups, suggesting that its finer crystalline microstructure provides greater resistance to wear. IPS e.max CAD groups, particularly in HT, displayed higher post-aging roughness, reinforcing the relationship between translucency modification, microstructure, and surface stability. These results are consistent with Özdemir et al. (2018), who reported that repeated firings increased surface roughness in lithium disilicate ceramics [33], and with Labban et al. (2021), who demonstrated that thermocycling and brushing abrasion significantly alter both surface texture and mechanical strength [34]. Meng et al. (2021) further supported the notion that material-specific microstructures determine resistance to simulated wear [35]. The consistent increase in Ra across all groups leads to rejection of the second hypothesis.

It is important to note that the brushing parameters used in the present study were selected to approximate the effects of five years of clinical use, based on established in vitro aging protocols. Previous investigations have estimated that an individual performs approximately 10,000 to 20,000 brushing strokes per year, depending on brushing frequency, technique, and applied force. Accordingly, a total of about 150,000 cycles under a standardized 200 g load has been widely adopted to simulate five years of toothbrushing in laboratory settings [36,37,38]. The present simulation therefore provides a realistic and reproducible representation of long-term mechanical wear under controlled conditions. Nevertheless, it should be acknowledged that in vitro brushing tests cannot fully replicate the complex intraoral environment, where factors such as saliva composition, pH fluctuations, and patient-specific habits also influence surface degradation over time.

Fracture resistance was also significantly affected by both translucency and ceramic type. LT ceramics generally outperformed their HT counterparts, and among all groups the highest values were observed in one LT ceramic, which reached 636 N. Comparisons within the same translucency level confirmed significant differences across materials, reflecting variations in formulation and crystallization behavior. These results indicate that translucency modifications, while beneficial for optical performance, may compromise mechanical behavior. Alsharawi et al. (2022) further emphasized that increased translucency in lithium disilicate is achieved through changes in the glassy matrix, which can reduce flexural strength, reinforcing the microstructure—mechanical property relationship [39]. In contrast, Akl et al. (2025) found no significant differences in flexural strength between different lithium disilicate systems or translucency levels; however, that study did not incorporate thermocycling or brushing simulation, limiting its conclusions to baseline conditions [40]. The present findings suggest that without environmental aging, important differences in ceramic performance may remain undetected.

Taken together, the results highlight a trade-off between surface smoothness and mechanical strength. LT ceramics exhibited smoother and more stable surfaces following aging, while some LT formulations also achieved the highest fracture resistance values. HT ceramics, although esthetically advantageous due to their greater translucency, tended to show higher surface degradation and comparatively lower strength. From a clinical perspective, these findings suggest that LT lithium disilicate ceramics may be preferred in situations requiring enhanced surface durability and long-term smoothness, whereas ceramics with superior fracture resistance may be more suitable for posterior load-bearing restorations.

The observed variations in surface roughness and fracture resistance among the evaluated lithium disilicate ceramics can be attributed to differences in sintering behavior and microstructural evolution. Sintering parameters, including firing temperature and holding time, play a crucial role in determining crystal morphology, size, and distribution within the glassy matrix [25]. Optimized sintering promotes uniform crystallization, enhancing mechanical integrity, whereas excessive temperature or prolonged firing can induce grain coarsening, increase surface roughness, and compromise esthetics. Moreover, intraoral aging factors such as thermocycling and simulated toothbrushing impose thermal and mechanical stresses that can degrade surface quality and facilitate microcrack initiation [24,26,41]. These mechanistic considerations help explain the greater degradation observed in HT ceramics and highlight the importance of controlling sintering and aging effects to maintain long-term performance. This confirms that surface roughness and fracture resistance are strongly affected by both sintering parameters and intraoral aging, underscoring the need to select and process each material according to its manufacturer-specific protocol and clinical indication.

To place these findings in a broader context, it is useful to compare them with other contemporary lithium disilicate ceramics, including zirconia-reinforced variants. Zirconia-reinforced lithium silicate ceramics (ZLS), such as Vita Suprinity, incorporate fine zirconia particles within a lithium silicate matrix, resulting in improved fracture toughness, flexural strength, and hardness compared to conventional lithium disilicate ceramics [16]. However, the inclusion of zirconia can influence translucency, and studies have shown that ZLS may exhibit reduced translucency compared to high-translucency lithium disilicate variants, potentially affecting esthetic outcomes [42]. Therefore, while zirconia reinforcement offers enhanced mechanical properties, it may necessitate trade-offs in optical performance. In our study, the differences observed between IPS e.max CAD and Amber Mill can be attributed to variations in their microstructural composition and crystal morphology. Again, these findings underscore the importance of balancing mechanical strength and translucency in the selection of dental ceramics, depending on the specific clinical requirements.

The current findings should be interpreted with caution as this investigation was limited to in vitro testing and therefore cannot fully reproduce the complexity of the intraoral environment, where saliva composition, pH fluctuations, enzymatic activity, and variable occlusal forces may further influence ceramic performance. Only two lithium disilicate systems were evaluated, which restricts the generalizability of the results to other glass-ceramic materials. Additionally, the aging regimen included thermocycling and brushing simulation but did not incorporate chewing or mechanical fatigue loading, limiting extrapolation to functional masticatory conditions. Surface roughness was assessed using only the Ra parameter, representing the arithmetic mean of surface deviations. Although Ra is widely used for comparative analysis, it does not fully describe the complex topography of ceramic surfaces. Different surface profiles may exhibit similar Ra values despite distinct morphological features. Future studies should therefore incorporate additional roughness parameters (e.g., Rq, Rz) or three-dimensional metrics (e.g., Sa, Sz) to provide a more comprehensive understanding of surface characteristics. Finally, combined aging protocols and long-term clinical trials are necessary to validate these findings and better predict the clinical performance of lithium disilicate ceramics.

## 5. Conclusions

Sintering parameters and artificial aging significantly affected the surface roughness and fracture resistance of lithium disilicate ceramics. Surface roughness increased after aging, and LT groups generally exhibited smoother surfaces and higher fracture resistance than HT counterparts, with fracture resistance ranging from 546 N to 636 N depending on translucency and brand. All investigated materials became rougher following thermocycling and brushing. These findings highlight the importance of optimizing sintering protocols for each commercial lithium disilicate system to achieve the best balance between esthetic quality and mechanical durability in clinical practice.

### Clinical Significance

Material selection should balance esthetics with mechanical performance. Low-translucency (LT) lithium disilicate offers superior surface durability and higher resistance to crack propagation, making it particularly suitable for posterior, load-bearing restorations, where mechanical demands are greatest. High-translucency (HT) ceramics, which provide improved light transmission and esthetic outcomes, may be more appropriate for anterior restorations, where esthetic considerations are paramount and occlusal forces are lower.

## Figures and Tables

**Table 1:** A summary of the different materials used in the investigation.

Test Group	Material	Brand Name	Composition	Level of Translucency
AMB_LT	Presintered lithium disilicate	Amber Mill (HassBio, Gangneung-si, Gangwon-do, Korea)	**Precrystallized state:** 44.9% LiSi_2_O_5_ 39.9% glass Crystallized state: 46.1% LiSi_2_O_5_ 33.7% glass	Low
AMB_HT				High
ECAD_LT	Presintered lithium disilicate	IPS e.max CAD (Ivoclar Vivadent, Schaan, Liechtenstein)	**Precrystallized state:** 32% LiSi_2_O_3_ 62% glass Crystallized state: 59% LiSi_2_O_5_ 33% glass	Low
ECAD_HT				High

**Table 2:** Manufacturer-recommended crystallization protocols for the different test groups.

Test Group	Crystallization Protocol	Predrying	Heating Rate (°C/min)	Temp 1 (°C)/Hold	Temp 2 (°C)/Hold
AMB_LT	One-step	400 °C	---	815 °C/15 min + 21.5 min vacuum	---
AMB_HT	One-step	400 °C	----	840 °C/22.2 min	----
ECAD_LT	Two-step	403 °C for 6 min	60 °C/min to 770 °C/10 min	30 °C/min to 850 °C/10 min	Cool to 700 °C
ECAD_HT	Two-step	403 °C for 6 min	60 °C/min to 770 °C/10 min	30 °C/min to 850 °C/10 min	Cool to 700 °C

**Table 3:** Mean surface roughness values (μm) before and after thermocycling and toothbrushing.

Test Group	Baseline Ra (Mean μm ± SD)	Post-Aging Ra (Mean μm ± SD)	*p*-Value
AMB_LT	0.24 ± 0.01	0.33 ± 0.01	<0.001 *
AMB_HT	0.43 ± 0.05	0.51 ± 0.07	<0.001 *
ECAD_LT	0.38 ± 0.05	0.43 ± 0.06	<0.001 *
ECAD_HT	0.49 ± 0.01	0.58 ± 0.01	<0.001 *

Ra: Surface roughness; SD: standard deviation. * indicates statistically significant differences.

**Table 4:** Mean fracture load values (N).

Test Group	Post-Aging Fracture Load (Mean N ± SD)	Significant Differences
AMB_LT	568.0 ± 14.73	a
AMB_HT	546.3 ± 21.90	b
ECAD_LT	566.4 ± 10.65	b
ECAD_HT	636.0 ± 8.29	c

Different superscript letters (a–c) indicate statistically significant differences among groups (*p* ≤ 0.05); *a* represents the highest and *c* the lowest mean value.

## Data Availability

The original contributions presented in the study are included in the article, further inquiries can be directed to the corresponding author.

## References

[1] Zhang Y., Kelly J.R. (2017). Dental Ceramics for Restoration and Metal Veneering. Dent. Clin. North Am..

[2] Zarone F., Di Mauro M.I., Ausiello P., Ruggiero G., Sorrentino R. (2019). Current status on lithium disilicate and zirconia: A narrative review. BMC Oral Heal..

[3] Rekow E.D., Silva N.R.F.A., Coelho P.G., Zhang Y., Guess P., Thompson V.P. (2011). Performance of Dental Ceramics: Challenges for Improvements. J. Dent. Res..

[4] da Silva L.H., de Lima E., Miranda R.B.d.P., Favero S.S., Lohbauer U., Cesar P.F. (2017). Dental ceramics: A review of new materials and processing methods. Braz. Oral Res..

[5] Deany I.L. (1996). Recent advances in ceramics for dentistry. Crit. Rev. Oral Biol. Med..

[6] Kelly J.R., Benetti P. (2011). Ceramic materials in dentistry: Historical evolution and current practice. Aust Dent J..

[7] Chen Y., Yeung A.W., Pow E.H., Tsoi J.K. (2021). Current status and research trends of lithium disilicate in dentistry: A bibliometric analysis. J. Prosthet. Dent..

[8] Culp L., McLaren E.A. (2010). Lithium disilicate: The restorative material of multiple options. Compend. Contin. Educ. Dent..

[9] Pieger S., Salman A., Bidra A.S. (2014). Clinical outcomes of lithium-disilicate single crowns and partial fixed dental prostheses: A systematic review. J Prosthet Dent..

[10] Zarone F., Ferrari M., Mangano F.G., Leone R., Sorrentino R. (2016). Digitally Oriented Materials”: Focus on Lithium-disilicate Ceramics. Int J Dent..

[11] Gehrt M., Wolfart S., Rafai N., Reich S., Edelhoff D. (2012). Clinical results of lithium-disilicate crowns after up to 9 years of service. Clin. Oral Investig..

[12] Ii R.G. (2022). Ceramics overview. Br. Dent. J..

[13] Al-Johani H., Haider J., Satterthwaite J., Silikas N. (2024). Lithium Silicate-Based Glass Ceramics in Dentistry: A Narrative Review. Prosthesis.

[14] Rinke S., Rödiger M., Ziebolz D., Schmidt A.-K. (2015). Fabrication of Zirconia-Reinforced Lithium Silicate Ceramic Restorations Using a Complete Digital Workflow. Case Rep. Dent..

[15] Taha D., Nour M., Zohdy M., El-Etreby A., Hamdy A., Salah T. (2019). The Effect of Different Wax Pattern Fabrication Techniques on the Marginal Fit of Customized Lithium Disilicate Implant Abutments. J. Prosthodont..

[16] Elsaka S.E., Elnaghy A.M. (2016). Mechanical properties of zirconia reinforced lithium silicate glass-ceramic. Dent. Mater..

[17] Mörmann W.H. (2006). The evolution of the CEREC system. J. Am. Dent. Assoc..

[18] Lubauer J., Belli R., Peterlik H., Hurle K., Lohbauer U. (2022). Grasping the Lithium hype: Insights into modern dental Lithium Silicate glass-ceramics. Dent. Mater..

[19] Phark J.H., Duarte Jr S. (2022). Microstructural considerations for novel lithium-disilicate glass ceramics: A review. J Esthet Restor Dent..

[20] Lu Y., Piva A.M.O.D., Nedeljkovic I., Tribst J.P.M., Feilzer A.J., Kleverlaan C.J. (2023). Effect of glazing technique and firing on surface roughness and flexural strength of an advanced lithium disilicate. Clin. Oral Investig..

[21] Aktas B., Yalcin S., Albaskara M., Aytar E., Ceyhan G., Turhan Z.Ş. (2022). Effect of Er2O3 on structural, mechanical, and optical properties of Al2O3-Na2O-B2O3-SiO2 glass. J. Non-Crystalline Solids.

[22] de Kok P., Pereira G.K., Fraga S., de Jager N., Venturini A.B., Kleverlaan C.J. (2017). The effect of internal roughness and bonding on the fracture resistance and structural reliability of lithium disilicate ceramic. Dent. Mater..

[23] Essam N., Soltan H., Attia A. (2023). Influence of thickness and surface conditioning on fracture resistance of occlusal veneer. BMC Oral Heal..

[24] Sehovic E., Ioannidis A., Hämmerle C.H., Özcan M., Mühlemann S. (2022). Effect of tooth brush abrasion on the color, gloss and surface roughness of internally and externally stained monolithic ceramic materials. J. Prosthodont. Res..

[25] Lima K.d.C., Vivanco R.G., Rodrigues P.R.B., Caetano A.L.P., Pires-De-Souza F.d.C.P. (2023). Long-term effect of firing protocols on surface roughness and flexural strength of lithium disilicate glass-ceramic. Braz. Dent. J..

[26] Alencar-Silva F.J., Barreto J.O., Negreiros W.A., Silva P.G., Pinto-Fiamengui L.M.S., Regis R.R. (2019). Effect of beverage solutions and toothbrushing on the surface roughness, microhardness, and color stainability of a vitreous CAD-CAM lithium disilicate ceramic. J. Prosthet. Dent..

[27] Floriani F., Jabr B., Rojas-Rueda S., Garcia-Contreras R., Jurado C.A., Alshabib A. (2025). Surface Analysis of Lithium Disilicate Ceramics After Use of Charcoal-Containing Toothpastes. J. Funct. Biomater..

[28] (2015). Dentistry—Ceramic Materials.

[29] Li D., Zhou M., Zhang Y., Meng M., Li X., Lyu X., Qin B., Wang F., Zhang Z. (2024). Effects of heat pressing on microstructure and mechanical properties of lithium disilicate glass ceramics with different crystal morphology. Ceram. Int..

[30] Höland W., Schweiger M., Frank M., Rheinberger V. (2000). A comparison of the microstructure and properties of the IPS Empress 2 and the IPS Empress glass-ceramics. J Biomed Mater Res..

[31] Al Ben Ali A., Kang K., Finkelman M.D., Zandparsa R., Hirayama H. (2014). The effect of variations in translucency and background on color differences in CAD/CAM lithium disilicate glass ceramics. J Prosthodont..

[32] Harianawala H.H., Kheur M.G., Apte S.K., Kale B.B., Sethi T.S., Kheur S.M. (2014). Comparative analysis of transmittance for different types of commercially available zirconia and lithium disilicate materials. J. Adv. Prosthodont..

[33] Özdemiṙ H., Özdoğan A. (2018). The effect of heat treatments applied to superstructure porcelain on the mechanical properties and microstructure of lithium disilicate glass ceramics. Dent. Mater. J..

[34] Labban N., Al Amri M.D., Alnafaiy S.M., Alhijji S.M., Alenizy M.A., Iskandar M., Feitosa S. (2021). Influence of Toothbrush Abrasion and Surface Treatments on Roughness and Gloss of Polymer-Infiltrated Ceramics. Polymers.

[35] Meng M., Wang X., Li K., Deng Z., Zhang Z., Sun Y., Zhang S., He L., Guo J. (2021). Effects of surface roughness on the time-dependent wear performance of lithium disilicate glass ceramic for dental applications. J. Mech. Behav. Biomed. Mater..

[36] Heintze S.D., Forjanic M. (2005). Surface roughness of different dental materials before and after simulated toothbrushing in vitro. Oper. Dent..

[37] Mondelli R., Garrido L., Soares A., Rodriguez-Medina A., Mondelli J., de Lucena F., Furuse A. (2022). Effect of simulated brushing on surface roughness and wear of bis-acryl-based materials submitted to different polishing protocols. J. Clin. Exp. Dent..

[38] Ximinis E., Dionysopoulos D., Papadopoulos C., Tournavitis A., Konstantinidis A., Naka O. (2023). Effect of tooth brushing simulation on the surface properties of various resin-matrix computer-aided design/computer-aided manufacturing ceramics. J. Esthet. Restor. Dent..

[39] Alsharawi T., Abdal Sadek H.M., Morsy T.S.E. (2022). Evaluation of translucency and biaxial flexural strength of different ceramic materials. Al-Azhar J Dent Sci.

[40] Akl M.A., Dashtti H., Akl J., Zheng F. (2025). Effect of crystallization temperature on the flexural strength of lithium disilicate glass ceramics. J. Prosthodont..

[41] Mahrous A.A., Alhammad A., Alqahtani F., Aljar Y., Alkadi A., Taymour N., Alotaibi A., Akhtar S., Gad M.M. (2023). The Toothbrushing Effects on Surface Properties and Color Stability of CAD/CAM and Pressable Ceramic Fixed Restorations—An In Vitro Study. Materials.

[42] Potdukhe S., Iyer J., More A. (2024). Effect of Artificial Aging on Translucency of Zirconia Reinforced Lithium Silicate and Lithium Disilicate Ceramics: A Systematic Review. Eur. J. Prosthodont. Restor. Dent..

